# Plasma HIV Viral Rebound following Protocol-Indicated Cessation of ART Commenced in Primary and Chronic HIV Infection

**DOI:** 10.1371/journal.pone.0043754

**Published:** 2012-08-31

**Authors:** Elizabeth Hamlyn, Fiona M. Ewings, Kholoud Porter, David A. Cooper, Giuseppe Tambussi, Mauro Schechter, Court Pedersen, Jason F. Okulicz, Myra McClure, Abdel Babiker, Jonathan Weber, Sarah Fidler, on behalf of the INSIGHT SMART and SPARTAC Investigators

**Affiliations:** 1 Kings College Hospital National Health Service Foundation Trust, London, United Kingdom; 2 Medical Research Council Clinical Trials Unit, London, United Kingdom; 3 The National Centre in HIV Epidemiology & Clinical Research and the University of New South Wales, Sydney, Australia; 4 Vaccine and Immunotherapy Research Centre, San Raffaele Scientific Institute, Milan, Italy; 5 Universidade Federal do Rio de Janeiro, Rio de Janeiro, Brazil; 6 Odense University Hospital, Odense, Denmark; 7 Infectious Disease Clinical Research Program, Uniformed Services University of the Health Sciences, Bethesda, Maryland, United States of America; 8 San Antonio Military Medical Center, Fort Sam Houston, Texas, United States of America; 9 Imperial College, London, United Kingdom; Ghent University, Belgium

## Abstract

**Objectives:**

The magnitude of HIV viral rebound following ART cessation has consequences for clinical outcome and onward transmission. We compared plasma viral load (pVL) rebound after stopping ART initiated in primary (PHI) and chronic HIV infection (CHI).

**Design:**

Two populations with protocol-indicated ART cessation from SPARTAC (PHI, n = 182) and SMART (CHI, n = 1450) trials.

**Methods:**

Time for pVL to reach pre-ART levels after stopping ART was assessed in PHI using survival analysis. Differences in pVL between PHI and CHI populations 4 weeks after stopping ART were examined using linear and logistic regression. Differences in pVL slopes up to 48 weeks were examined using linear mixed models and viral burden was estimated through a time-averaged area-under-pVL curve. CHI participants were categorised by nadir CD4 at ART stop.

**Results:**

Of 171 PHI participants, 71 (41.5%) rebounded to pre-ART pVL levels, at a median of 50 (95% CI 48–51) weeks after stopping ART. Four weeks after stopping treatment, although the proportion with pVL≥400 copies/ml was similar (78% PHI versus 79% CHI), levels were 0.45 (95% CI 0.26–0.64) log_10_ copies/ml lower for PHI versus CHI, and remained lower up to 48 weeks. Lower CD4 nadir in CHI was associated with higher pVL after ART stop. Rebound for CHI participants with CD4 nadir >500 cells/mm^3^ was comparable to that experienced by PHI participants.

**Conclusions:**

Stopping ART initiated in PHI and CHI was associated with viral rebound to levels conferring increased transmission risk, although the level of rebound was significantly lower and sustained in PHI compared to CHI.

## Introduction

Long-term use of antiretroviral therapy (ART) in HIV-positive persons may be challenged by the need for high-level adherence, development of drug resistance, toxicities, and cost. Treatment strategies conferring durable virological control, whilst minimising ART exposure are highly desirable. With this goal in mind, strategic interruption of ART was the focus of several studies [1]–[3].

However, interruption of ART is no longer a recommended strategy [2] and the level of HIV plasma viral load (pVL) following ART stop has been shown to reach levels comparable to pre-treatment values [2]–[4], increasing onward transmission risk [5]. Inaccessible reservoirs of latently-infected resting memory CD4 T-cells are hypothesised to be the major source contributing to viraemia rebound after stopping ART [6], [7].

Recent research has shown the dramatic effect of ART to prevent onward viral transmission [8], and mathematical models predict that it may potentially be possible to eliminate HIV infection at a population level with universal treatment coverage for all HIV-positive individuals, irrespective of CD4 count [9]. However, although not recommended, consideration of the potential impact of individuals choosing to stop ART could be considerable, and data are needed on subsequent viral rebound to better inform future transmission models. Furthermore, final results from SPARTAC suggested that ART initiated in primary HIV infection (PHI) was associated with a change in pVL set-point out to 60 weeks after stopping therapy [10] whilst the SMART trial reported that interruption of ART in chronic infection (CHI) was associated with an increased risk of all-cause mortality

The level of viral rebound following interruption of ART commenced in at different stages of HIV infection is, therefore, highly relevant from both a clinical and public health perspective and warrants further investigation.

We, therefore, wanted to compare the pVL changes observed after cessation of ART initiated in chronic HIV infection with those in PHI by comparing viral rebound between individuals enrolled in two protocol-indicated ART interruption studies; SPARTAC and SMART.

## Methods

### Ethics statement

The SPARTAC trial was approved by the following authorities: Medicines and Healthcare products Regulatory Agency (UK), Ministry of Health (Brazil), Irish Medicines Board (Ireland), Medicines Control Council (South Africa), and the Uganda National Council for Science and Technology (Uganda). It was also approved by the following ethics committees in the participating countries: Central London Research Ethics Committee (UK), Hospital Universitário Clementino Fraga Filho Ethics in Research Committee (Brazil), Clinical Research and Ethics Committee of Hospital Clinic in the province of Barcelona (Spain), The Adelaide and Meath Hospital Research Ethics Committee (Ireland), University of Witwatersrand Human Research Ethics Committee, University of Kwazulu-Natal Research Ethics Committee and University of Cape Town Research Ethics Committee (South Africa), Uganda Virus Research Institute Science and ethics committee (Uganda), The Prince Charles Hospital Human Research Ethics Committee and St Vincent's Hospital Human Research Ethics Committee (Australia), and the National Institute for Infectious Diseases Lazzaro Spallanzani, Institute Hospital and the Medical Research Ethics Committee, and the ethical committee Of the Central Foundation of San Raffaele, MonteTabor (Italy). The INSIGHT SMART trial was approved by the University of Minnesota institutional review board. All participants signed a written informed consent.

### Study populations

Viral dynamics following treatment interruption were compared using data from SPARTAC and SMART participants. SPARTAC is an international RCT comparing no therapy, 12-week ART, or 48-week ART initiated within a maximum of 6 months from the last documented HIV negative test date. The primary outcome measure was time to confirmed CD4 cell count <350 cells/mm^3^, or the initiation of long-term therapy. PHI was identified according to the trial protocol. The trial recently reported a significant difference in time to the primary endpoint for the 48-week, but not the 12-week, ART arm compared to no therapy, although not significantly longer than the time already spent on therapy [10]. SMART is an international RCT which compared a CD4-guided strategy of planned treatment interruptions versus continuous ART in chronically HIV-infected individuals. Eligible participants with CD4>350 cells/mm^3^ were randomised to either a Drug Conservation (DC) or Viral Suppression (VS) arm. Enrolment was stopped on 11^th^ January 2006 and participants in the DC arm were recommended to re-initiate ART as interim results clearly indicated superiority of the VS arm [2].

Individuals were included in this analysis if they underwent protocol-indicated ART cessation, i.e. on ART at time of randomisation to the DC arm in SMART (hereafter, the chronically-infected population) or randomised to one of the two treatment arms in SPARTAC (hereafter, the PHI population), and had a CD4 cell count and pVL available at the time of ART stop. Subsequent pVL measurements were scheduled at 4, 12, 24, 36, 48 and 4, 8, 16, 32, 40, 48 weeks after ART stop in SPARTAC and SMART respectively. pVLs were determined locally; for included participants, 40, 59, <1 and <1% from SPARTAC and 20, 74, 4 and 2% from SMART were measured using bDNA, PCR, NASBA and other assays, respectively.

### Statistical methods

We examined the time following ART stop for pVL to reach pre-ART levels in the PHI population, using survival methods. We then compared pVL levels at 4 weeks after stopping ART in the PHI participants, and up to 48 weeks afterwards, with those in chronically-infected individuals. Using linear and ordered logistic regression, respectively, we examined differences in absolute levels and in the proportions with pVL<400, 400–3499, 3500–9999, 10,000–49,999 or ≥50,000 copies/ml at 4 weeks after ART stop [5]. Using linear mixed models, we examined differences in pVL levels and slopes over 4–48 weeks after ART stop, and estimated predicted pVLs at 4 and 36 weeks after ART stop for representative PHI and chronically-infected participants (male infected through sex with men, aged 40 years and with CD4 600 cells/mm^3^ at ART stop). We estimated the complete viral burden, through a time-averaged area–under-pVL curve, over the whole period after stopping ART. We then categorised the chronically-infected participants according to their nadir CD4 count at ART stop in order to assess whether any differences over 48 weeks between the populations could be explained solely by nadir CD4.

Follow-up began from the date of first stopping all drugs in the ART regimen and was censored at the last pVL measurement, the 48 week visit, when ART was re-initiated, or 11^th^ January 2006 for SMART participants, whichever was earliest.

We restricted analyses to participants who had suppressed pVL to <400 copies/ml at the time of ART stop, as this was the limit of the least sensitive assay used across both trials. pVL data were log_10_-transformed and values <400 copies/ml were treated as  = 400 copies/ml for all participants to avoid confounding by trial, as a greater proportion of pVLs were measured using this detection limit in the PHI compared to chronically-infected participants (13 versus 6% <400 copies/ml and 9 vs. 16%, respectively, <50 copies/ml). CD4 and pVL at ART stop were defined by those closest to ART stop (up to 24 and 12 weeks before, respectively, and no more than 2 weeks after). Subsequent pVLs were defined by those closest to the scheduled visits (allowing a +/−2 week window around the week 4 and 6 visits and a +/−4 week window around subsequent visits). Pre-ART pVLs in PHI participants were estimated as the mean of all available pVLs before ART initiation (9%, 85%, 5%, 1% participants had 1, 2, 3 and 4 pre-ART pVLs available, respectively). We also restricted analyses to include sexually-infected individuals only as few were from other risk groups in the PHI population.

Models were adjusted for the effect of sex/risk group (sex between men (MSM), heterosexual men or heterosexual women), age and CD4 at ART stop. No adjustments were made for time on ART, as this is confounded by duration of infection and, therefore, by trial, nor for ART class (also confounded by trial). Using only data from the chronic population, however, we investigated the possible effect of ART class on viral rebound. We also investigated whether there were differences in the effects of participant characteristics at ART stop for PHI compared to chronically-infected participants, and CD4 nadir up to ART stop, where appropriate, using interactions.

As SMART participants were enrolled into the trial with prevalent HIV infection, duration of HIV infection and time since first initiation of ART may not have been known and so reported values should be regarded as best estimates. In particular, the duration of infection is based on first known HIV positive result, therefore, the intervals are likely to be underestimates.

## Results

### Description of the population

Of the 243 SPARTAC participants randomised to one of the two treatment arms, 16 were excluded because they did not initiate ART (n = 5), did not stop ART (n = 6), were on ART for <15 days (n = 4) or did not have a pVL at ART stop (n = 1). Of the 2290 SMART participants on ART at the time of randomisation to the DC arm, 256 were excluded because they did not stop ART (n = 42, 25 of whom were randomised in the month prior to 11^th^ January 2006), stopped ART after 11^th^ January 2006 (n = 20), did not have a pVL at ART stop (n = 1) or did not have any subsequent pVLs after stopping ART (n = 193, 153 of whom only stopped in the month prior to 11^th^ January 2006). Additional exclusions were as follows: 43 SPARTAC and 384 SMART participants with pVL≥400 copies/ml at ART stop, 125 SMART participants with reported risk group IDU, and 2 SPARTAC and 75 SMART participants with other/unknown route of HIV transmission. Therefore, 182 PHI and 1450 chronically-infected participants were included in our analyses.

Participant demographics, ART exposure and CD4 at time of treatment discontinuation are shown in Table 1. Compared to those chronically-infected, PHI participants were younger (median 34 versus 44 years), more likely to be female (33% versus 24%), had considerably less ART exposure (6% versus 44% ever exposed to ≥3 drug classes) and were more likely to be on a protease inhibitor regimen at the time of ART stop (94% versus 36%). At ART stop, median CD4 was slightly higher among PHI compared to chronically-infected participants (707 versus 646 cells/mm^3^). Among chronically-infected participants, 76% had nadir CD4<350 cells/mm^3^. Five-hundred and fifty-nine (39%) chronically-infected participants were censored on 11^th^ January 2006 due to discontinuation of the SMART DC arm. A further 17 (9%) PHI and 463 (32%) chronically-infected participants were censored before their 48 week visit due to ART re-initiation. The median (IQR) follow-up was 48 (45, 49) and 27 (12, 43) weeks for the PHI and chronically-infected participants, respectively, and the median (IQR) number of RNA measurements included per individual was 6 (5, 6) and 4 (3, 6), respectively.

**Table 1:** Participant characteristics at ART stop.

	CHRONIC HIV INFECTION	PHI (SPARTAC) N = 182
	(SMART) N = 1450	
Sex, female (n, %)	350 (24%)	60 (33%)
Age, years (median, IQR)	44 (38, 51)	34 (28, 42)
HIV exposure (n, %)		
Sex between men	890 (61%)	114 (63%)
Sex between men & women (male)	222 (15%)	8 (4%)
Sex between men & women (female)	338 (23%)	60 (33%)
Time since first diagnosed HIV positive, months	96 (60, 144)	6 (4, 13)
(median, IQR)		
Number of ART drugs, ever (median, IQR)	5 (4, 7)	3 (3, 3)
Number of ART classes, ever (n, %)		
1	48 (3%)	0 (0%)
2	763 (53%)	171 (94%)
≥3	639 (44%)	11 (6%)
Estimated time on therapy, months (median, IQR)	72 (48, 96)	3 (3, 11)
ART type at stop (n, %)		
NNRTI based	674 (46%)	8 (4%)
PI based	521 (36%)	171 (94%)
3 NRTI	132 (9%)	2 (1%)
3 class	84 (6%)	0 (0%)
NRTI sparing	3 (<1%)	0 (0%)
Suboptimal ART	36 (2%)	1 (1%)
Nadir CD4 count up to ART stop, cells/mm^3^	230 (132, 340)	-
(median, IQR; below: n, %)		
<200	592 (41%)	-
200–349	515 (36%)	-
350–499	226 (16%)	-
≥500	117 (8%)	-
CD4 count at ART stop, cells/mm^3^ * (median,	646 (495, 848)	707 (586, 919)
IQR; below: median, IQR by nadir CD4)		
nadir CD4<200 cells/mm^3^	568 (456, 724)	-
nadir CD4 200–349 cells/mm^3^	618 (494, 784)	-
nadir CD4 350–499 cells/mm^3^	806 (646, 993)	-
nadir CD4≥500 cells/mm^3^	948 (784, 1176)	-

* Closest up to 24 weeks before ART stop. NB: 2 chronically-infected participants had CD4 count <350 cells/mm^3^ at ART stop (contrary to SMART inclusion criteria), but both were measured on the day of ART stop and both participants had previous CD4 count >350 cells/mm^3^ within the previous 6 weeks.

### Time to pVL reaching pre-ART levels in PHI participants

Among the PHI participants, the median (IQR) pre-ART pVL was 4.5 (3.9, 5.1) log_10_ copies/ml. Eleven participants had pre-ART pVL<400 copies/ml and were, therefore, omitted from the analyses of estimating time to reaching pre-ART pVL. Nine participants, who had higher median pre-ART pVL (5.5 log_10_ copies/ml), were censored before reaching pre-ART levels due to ART re-initiation (one at 5 weeks and the remainder ≥25 weeks after ART stop). A total of 71 (42% of 171) participants were observed to rebound to pre-ART pVL levels, at a median of 50 (95% CI 48, 51) weeks. A quarter of participants had rebounded to pre-ART levels by 15 (95% CI 12, 26) weeks.

### pVL rebound after ART stop by PHI versus chronically-infected participants

At 4 weeks after ART stop, the proportions with pVL<400 copies/ml were similar in the two groups (Table 2), but median pVL levels were significantly lower among PHI compared to chronically-infected participants (unadjusted median 3.7 versus 4.4 log_10_ copies/ml, respectively; adjusted pVL 0.45 (95% CI 0.26, 0.64) log_10_ copies/ml lower, p<0.001; Table 3). Higher CD4 cell count at ART stop was weakly associated with lower week 4 pVL, and persons infected through heterosexual contact had lower week 4 pVL compared to MSM, but with no evidence that this effect differed between the PHI and chronically-infected groups (p = 0.4). There was no association between age at ART stop and week 4 pVL (p = 0.6).

**Table 2:** pVL levels at 4 weeks after ART stop by PHI versus chronically-infected participants.

pVL, copies/ml	Chronic HIV infection	PHI (SPARTAC) N = 156*
	(SMART) N = 1327*	
<400	284 (21%)	35 (22%)
400–3499	161 (12%)	34 (22%)
3500–9999	110 (8%)	36 (23%)
10,000–49,999	235 (18%)	25 (16%)
≥50,000	537 (40%)	26 (17%)

Values are n (%). pVL = plasma viral load.

Adjusted p-value from ordered logistic regression <0.001.

* Of participants with a week 4 pVL available.

**Table 3:** Factors associated with pVL level (log_10_ copies/ml) at 4 weeks after ART stop (from adjusted linear regression model*).

	Coefficient (95% CI)	P
PHI, versus chronically-infected	−0.45 (−0.64, −0.26)	<0.001
Age at ART stop, per 10 years	0.01 (−0.04, 0.07)	0.6
Sex/risk group, vs men infected through sex with men		<0.001
Male, infected through sex with women	−0.14 (−0.30, 0.03)	
Female, infected through sex with men	−0.33 (−0.47, −0.20)	
CD4 count at ART stop, per 100 cells/mm^3^	−0.02 (−0.04, 0.002)	0.08
Constant**	4.26 (4.18, 4.34)	-

CI = confidence interval. pVL = plasma viral load. Coefficients are interpreted as the value of log_10_ copies/ml lower pVL for a negative sign, and higher for a positive sign.

* Adjusted for factors in the table.

** Mean week 4 pVL for a chronically-infected male infected through sex with men, aged 40 years and with CD4 600 cells/mm^3^ at ART stop.

Considering only the chronically-infected participants, pVL was significantly higher at 4 weeks after ART stop for those on PI-based or triple NRTI regimens, compared to those on NNRTI-based regimens (0.69 [0.56,0.81] and 0.58 [0.38,0.79] log_10_ copies/ml, respectively). There was no evidence of a difference for other highly-active or suboptimal regimens, compared to NNRTI-based regimens (0.36 [−0.01,0.74] and 0.17 [−0.07,0.42] log_10_ copies/ml, respectively.

**Figure 1:**
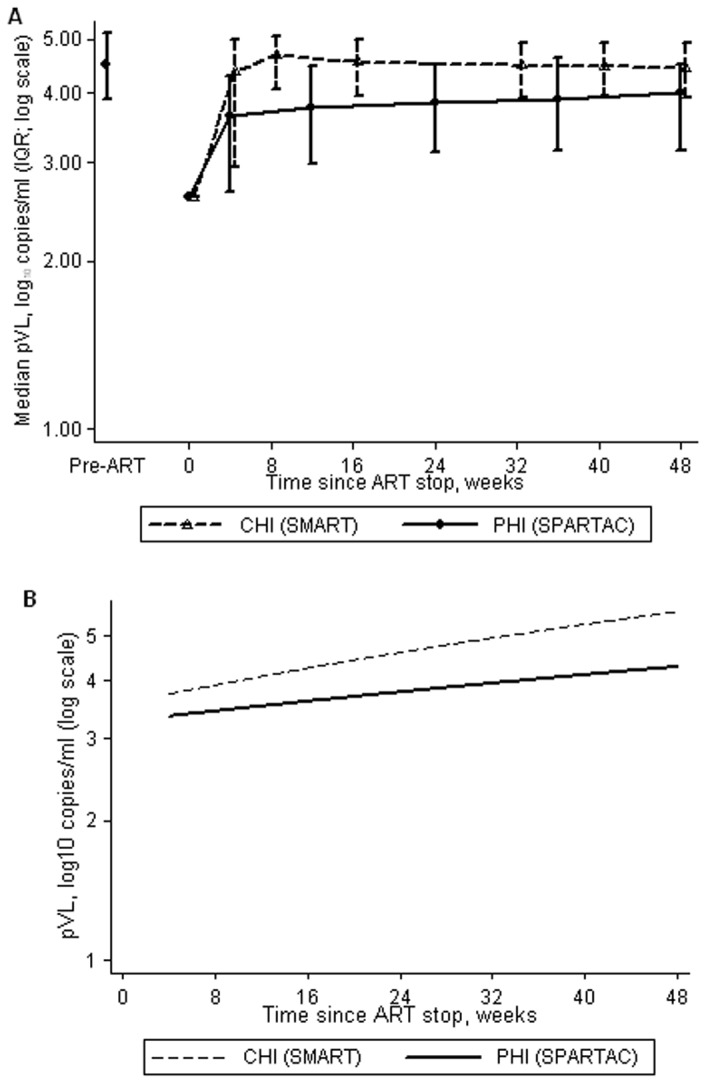
pVL after ART stop in primary (PHI) and chronic HIV infection (CHI). a. median (IQR) pVL up to 48 weeks after ART stop. b. predicted pVL over 4–48 weeks after ART stop, based on a representative participant (male infected through sex with men, aged 40 years and with CD4 count 600 cells/mm^3^ at ART stop; values in brackets are the 95% CI). CI = confidence intervals, IQR = interquartile range, pLV = plasma viral load.

Over 48 weeks after ART stop, median pVL remained lower in individuals with PHI compared to chronically-infected participants (Figure 1a), with evidence to suggest that chronically-infected participants rebounded more rapidly than PHI participants (i.e. had steeper slope; adjusted p<0.0001; Figure 1b). Predicted pVLs for representative participants are given in Figure 1b.

The median (IQR) viral burden was 1.12 (0.56, 1.69) and 1.55 (1.03, 1.99) log_10_ copies/ml amongst PHI and chronically-infected participants, respectively. After adjustment, viral burden was, on average, 0.28 (95% CI 0.17, 0.39) log_10_ copies/ml lower for PHI versus chronically-infected participants (p<0.001).

The associations between longer-term pVL rebound and sex/risk group, CD4 cell count and age at ART stop were qualitatively similar as those for the week 4 pVL rebound (results not shown).

### pVL rebound after ART stop, categorising the chronically-infected participants by nadir CD4 count

**Figure 2:**
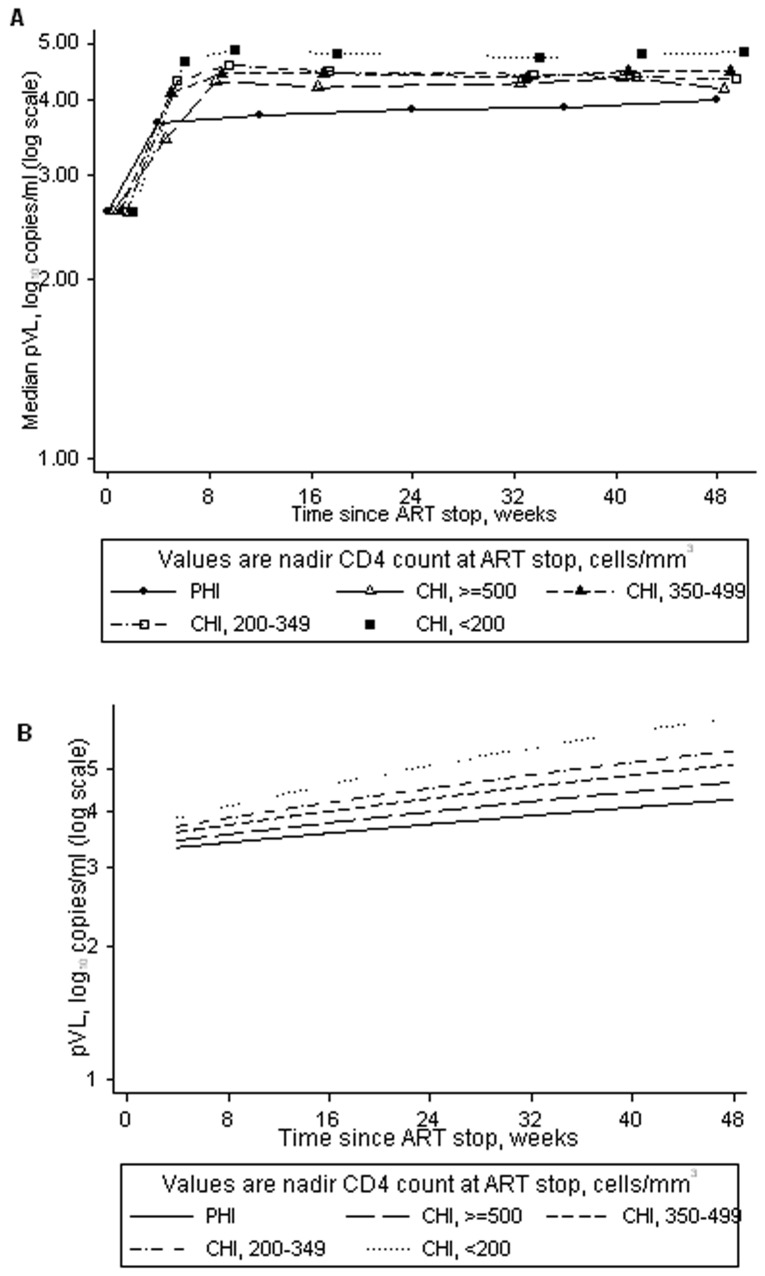
pVL after ART stop in primary (PHI) and chronic HIV infection (CHI), with CHI participants categorised by nadir CD4 count. a. median (IQR) pVL up to 48 weeks after ART stop. b. predicted pVL over 4–48 weeks after ART stop, based on a representative participant (male infected through sex between men, aged 40 years and CD4 count 600 cells/mm^3^ at ART stop; values in brackets are the 95% CI). CI = confidence intervals, IQR = interquartile range, pLV = plasma viral load.

Over 4–48 weeks after ART stop, pVL remained significantly higher in chronically-infected participants with nadir CD4<500 cells/mm^3^, compared to PHI participants. Lower CD4 nadir was associated with faster rebound (Figures 2a and 2b). For every 8 weeks the pVL in PHI participants increased, on average, by 0.17 (95% CI 0.14, 0.20) log_10_ copies/ml compared to 0.15 (0.01, 0.29), 0.33 (0.21, 0.44), 0.46 (0.36, 0.57) and 0.71 (0.60, 0.81) log_10_ copies/ml in chronically-infected participants with nadir CD4≥500, 350–499, 200–349, and <200 cells/mm^3^, respectively. Predicted pVL levels for representative participants are given in Figure 2b.

We observed a similar relationship for viral burden: chronically-infected participants with a lower nadir CD4 had higher viral burden, compared with PHI participants (0.39 [95% CI 0.27, 0.51], 0.29 [0.18, 0.41] and 0.20 [0.06, 0.33] log_10_ copies/ml higher viral burden for nadir CD4<200, 200–349 and 350–499 cells/mm^3^ respectively), but there was no difference in viral burden between chronically-infected participants with CD4 nadir ≥500 cells/mm^3^ and PHI participants (0.04 [−0.12 to 0.20] log_10_ copies/ml).

The effects of sex/risk group and age at ART stop were similar to those for week 4 pVL rebound (results not shown). However, we found evidence of an interaction between nadir CD4 and CD4 cell count at stop (p = 0.04 and 0.0006 in the linear mixed model for pVL up to 48 weeks and the linear regression for viral burden, respectively). CD4 count 100 cells/mm^3^ higher at ART stop was associated with a higher viral burden of 0.05 (95% CI 0.03, 0.08) and 0.02 (−0.002, 0.05) log_10_ copies/ml for chronically-infected participants with nadir CD4<200 and 200–349 cells/mm^3^, respectively. Among chronically-infected participants with nadir CD4 350–499 or ≥500 cells/mm^3^, there was no evidence of such an association (viral burden 0.002 [−0.03, 0.04] and −0.01 [−0.05, 0.03] log_10_ copies/ml higher per 100 cells/mm^3^ higher CD4 at ART stop, respectively). Among PHI participants, there was evidence to suggest that higher CD4 at ART stop was associated with lower viral burden (0.04 [95% CI 0.003, 0.08] log_10_ copies/ml lower per 100 cells/mm^3^ higher CD4 at ART stop). The effect on viral burden of PHI versus chronic infection/nadir CD4 remained robust with or without adjustment for CD4 count at ART stop.

## Discussion

This is the first study to compare HIV pVL dynamics between PHI and chronically-infected individuals undergoing a protocol-indicated ART interruption. We observed that pVL rebound after stopping ART initiated in PHI was lower than that observed in chronic infection, at 4 weeks after treatment interruption, and this difference was sustained over 48 weeks of follow-up. In addition, the overall viral burden, as estimated by the area under the pVL by time curve, was significantly lower in PHI compared to chronically-infected participants.

Our findings support those from a smaller study which observed significantly shorter time to viral rebound following treatment interruption in participants who initiated treatment in PHI compared to chronic infection [11]. However, they did not consider the stage of infection prior to commencing therapy. We found that, as anticipated, when participants with chronic infection were stratified by nadir CD4 at time of stopping ART, lower nadir CD4 was associated with higher pVL after stopping therapy. Compared to PHI, viral rebound was higher in chronically-infected participants with nadir CD4<500 cells/mm^3^, but similar to levels experienced by those with nadir CD4≥500 cells/mm^3^. Interestingly, in chronically-infected participants with CD4 nadir <200 cells/mm^3^, higher CD4 count at ART stop was associated with subsequent higher viral burden. Thus, it could be hypothesised that the degree of viral rebound may be related to the degree of immune reconstitution occurring during ART, or to the number of CD4 target cells available for viral infection at ART stop [12].

The observed difference in virological impact of stopping ART in PHI versus chronic infection may reflect differences in viral reservoir size although no data were available from either trial on HIV reservoir size and we were, therefore, unable to directly examine this. A study of ART initiated during PHI found that 36 weeks of therapy reduced proviral HIV-1 DNA to levels comparable to those seen in long-term non-progressors whilst, although levels were also reduced in chronic infection, they remained significantly higher than in PHI and long-term non-progressors [13]. This was supported by others reporting evidence for decay of the reservoir in patients who initiated ART early in infection [14] and a significant reduction in its size in those initiating ART early, compared to chronic, infection [15]. However, others quantifying the viral reservoir in treated PHI participants reported that, although a reduction in reservoir size is observed after even short-course ART initiated in PHI, complete abolition of viral replication is not achieved and viral reservoir may be re-expanded even after short-term rebound of viraemia [16].

As the majority of studies examining short-course ART in PHI are observational in nature, the reason for starting or stopping therapy may be related to prognosis. In our analysis, the protocol-indicated ART cessation in both trial populations minimises the effect of this potential source of bias, although this study has some limitations. It was not possible to adjust for ART duration, which was longer for the chronically-infected compared to PHI participants, or for ART class. It is also possible that some SMART participants may have initiated ART in primary infection, although this information is not captured. Our analyses, restricted to the chronically-infected participants only, however, indicated that those previously on NNRTI-based regimens had lower week 4 pVL rebound compared to those on other regimens. Since a greater proportion of chronically-infected, compared to PHI participants, were previously on NNRTI-based regimens, adjustment for ART class would have only served to augment the differences reported here between the groups. Longitudinal analysis of both populations is also subject to bias due to informative censoring, in particular due to exclusion of data for individuals who re-initiated ART. However, since a higher proportion of chronically-infected compared to PHI participants reinitiated therapy (32% versus 9% before week 48), the results presented here are likely to be an underestimate of the difference between the two populations. Although pVL assays varied according to location, it is unlikely, given the pVL ranges in these analyses, that use of different commercial pVL assays would significantly affect the results.

In both trials, pVL was not measured until 4 weeks following treatment interruption. Although PHI participants were not observed to rebound to pre-ART pVL levels until a median of 50 weeks, the pVL levels may have been greater before week 4. Earlier and more frequent testing would give a better indication of immediate viral dynamics following ART cessation. The move in the HIV prevention field to explore a universal “test and treat” strategy [9], [17]–[18] is currently receiving much scientific and advocacy interest. Although mathematical models are encouraging, the effectiveness of such an approach will depend on sustained adherence to therapy. Transmission risk has been shown to be higher in those with pVL>1500 copies/ml [5], [19]. The data presented in our analysis show that, irrespective of disease stage and nadir CD4 count, the level of rebound viraemia on stopping ART in the vast majority of individuals reaches a level above which transmission can occur. Targeting individuals with ART during PHI could have a marked impact on HIV transmission [20], but it is crucial that strategies investigating the use of ART as transmission prevention examine the consequences of ART discontinuation and viral rebound on onward transmission. In addition to the impact of pVL on transmission, the sexual behaviour of those individuals critically impacts their transmission risk at a population level [21]. In the SMART trial, individuals did not reduce high-risk sexual behaviour despite treatment interruption and detectable pVL [22]. This was not investigated in SPARTAC. However, in a recent study looking at onward HIV transmission amongst 47 individuals treated in PHI who stopped ART, there were at least five new primary infection events originating from these persons within 16–61 weeks after stopping early ART [23].

This analysis provides estimates for the viral rebound following cessation of ART initiated in PHI or chronic infection, and may inform mathematical models evaluating the potential population effect of universal treatment on HIV incidence for individuals stopping ART. The demonstrated differences in viral load dynamics following ART cessation between PHI and chronic infection indicate that the consequences of treatment interruption may differ, potentially reflecting differences in immunological status, HIV activation and reservoir size. This analysis supports the necessity for sustained virological suppression to limit onward transmission risk if a “test and treat” approach is to deliver a sustained population level effect on HIV incidence.
